# Overall structural seismic damage rapid assessment method based on period and displacement response characteristics

**DOI:** 10.1038/s41598-022-23927-x

**Published:** 2022-11-11

**Authors:** Mingzhen Wang, Lin Gao, Zailin Yang

**Affiliations:** 1 College of Aerospace and Civil Engineering, Harbin Engineering University, Harbin, 150001 China; 2 College of Civil Engineering, Chongqing University of Arts and Sciences, Chongqing, 402160 China; 3 Beijing Key Lab of Earthquake Engineering and Structural Retrofit, Beijing University of Technology, Beijing, 100124 China

**Keywords:** Civil engineering, Mechanical engineering

## Abstract

The seismic damage state of building structure can be rapidly evaluated by coupling effect of structural displacement response and periodic characteristics. Firstly, the fundamental period calculation formula that adapts to the deformation pattern and distribution mode of horizontal seismic action for reinforced concrete frame structure is derived. Secondly, the seismic damage assessment standard of building structure considering period variation is established. Then, the seismic damage assessment method of building structure is constructed. Finally, the seismic damage example is used to verify the established evaluation method. The results show that the established research method has high accuracy and good engineering practicability.

## Introduction

Compared with displacement-based structural damage assessment method, coefficient-based method with consideration of structural vibration characteristics has obvious advantages of high evaluation efficiency and independent of manual judgment. When structural vibration characteristics are used to evaluate building damage, the commonly used parameters include period (or frequency), vibration mode, damping ratio, frequency response function, curvature mode, modal flexibility, modal strain energy, Ritz vector and residual stress vector. The four parameters of natural vibration period (or frequency), vibration mode, damping ratio and frequency response function can be obtained not only by dynamic characteristics test, but also by numerical simulation analysis of finite element model. For the four parameters of curvature mode, modal flexibility, modal strain energy, residual stress vector, they can only be obtained by numerical simulation analysis.

Affected by uncertain factors such as material properties, contact connection and boundary conditions, the established finite element model is difficult to accurately simulate the real situation of the building structure. Therefore, when the curvature mode, modal flexibility, modal strain energy, residual stress vector and other parameters are used to evaluate the building damage, the results are easy to deviate from the actual situation. With the development of test instruments and signal analysis tools, natural vibration period, vibration mode, damping ratio and other parameters can be obtained only by analyzing the actual measured structural response data. Moreover, compared with the structural dynamic characteristics such as vibration mode and damping ratio, the natural vibration period has the obvious characteristics of easy acquisition and high identification efficiency. Therefore, the natural vibration period is usually selected as the main parameter to quickly evaluate the overall damage of the structure.

In the process of establishing structural seismic damage assessment method based on natural vibration period, it is necessary to focus on two problems. (a) It should be established a fundamental period estimation formula which is simplexes and clear physical significance as well as have relatively accurate estimation results. (b) It should be constructed a functional relationship between fundamental period and structural seismic damage. For the above, the relevant researchers have conducted a lot of research and achieved certain research results.

Gilles et al.^1^ analyzed the theoretical basis of the seismic action calculation in the equivalent static method of NBCC standard, and researched the differences of base shear calculation results for different height buildings between using experience fundamental period formula and standard methodology. The results show that when the fundamental period is calculated by empirical formula for estimating results, the base shear of the structure is lowered by 3.5 times.

Sofi et al.^2^ focused on analyzing the mechanical principles and main characteristics of various fundamental period computational formulas, and analyzed the impact of masonry infilled wall, concrete or cement block partition wall on the fundamental period.

Sangamnerkar and Dubey^3^ analyzed 36 reinforced concrete frame structures with different underlying dimensions and shaft-spacing structures. And influencing factors on the fundamental period are researched such as the underlying width, the column cross-section size and the stiffness of the structural basis. The results show that the growth ratio of structural fundamental period is proportional to the underlying width growth.

Young and Adeli^4^ designed 12 eccentric cantilever steel frame structures with different heights, spans and spatial stiffness distributions, and used ETABS to analyze the fundamental periods of different structures. Comparing to ASCE7-10 formula, Raylei formula and ETABS analysis results, the recommendation fundamental period calculation formula for different types of eccentric cantilever steel frame structures are given.

Wang et al.^5^ analyzed 414 high-rise, super-high-rise reinforced concrete structures and mixed structures. The main influencing factors of the fundamental period are comprehensively analyzed, and the fundamental period calculation formula for high-rise building structure is fitted. And 15 shake table test data, 27 pulsation tests, wind test data and Chinese standardized calculation formula calculate results are used to verify the fitted formula. After the correction, the fundamental period calculation formula and the first three-order cycle ratio relationship for high-rise and supper-high-rise building are given.

Based on 90 fundamental period data of steel plate shear wall structures collected in literatures, Jiang et al.^6^ determined a new calculation formula according to the multi-freedom structure dynamic characteristics calculation theory. And the research formula is verified by the shake table tests.

In response to the shortcomings of the fundamental period of shear wall structures in the Indian seismic code, Mandanka et al.^7^ selected 23 irregular shear walls considering the stiffness regular with different planar dimension, structural height, shear wall size, etc., and analyzed the fundamental period of these buildings by using ETABS software. Based on the numerical simulation analysis data, a new fundamental period estimation formula for the stiffness irregular reinforced concrete shear wall structure is fitted considering influencing factors such as total structural height, structural width, and inertial moments.

Elfath and Elhout^8^ applied the Egyptian code to design 36 steel frame structures with different structural height, seismic intensity and elastic story-drift angle, and the change pattern of fundamental period was analyzed focusing on the changes of structural stiffness and height distribution. It is believed that the fundamental period of the bending frame structure is closely related to the seismic intensity and story-drift angle.

For the coupling relationship between the fundamental period and the structural seismic damage, the relevant researchers have been researched.

Eleftheriadou and Karabinis^9^ statistically analyzed the damage data of 164,135 buildings in the Parnitha 5.9 earthquake in 1999. The relationships between the range of different fundamental periods and the damage ratio of buildings corresponding to different damage levels have been focused on.

Based on 300,000 nonlinear seismic response time history analysis data, Katsanos and Sextos^10^ used the theory of elastoplastic response spectrum to study the calculation method of the period elongation of the building structure under the seismic damage state. Research results shows that the periodic elongation rate of damaged structures is significantly affected by the period of structural elasticity and the rate of structural stiffness degradation.

Sarno and Amiri^11^ established a nonlinear single-degree-of-freedom structural system for reinforced concrete structures, taking into account structural factors such as structural ductility coefficient and stiffness degradation rate, and used OpenSees software input to consider the ground motion of the main aftershock sequence for nonlinear time history analysis. The relationship between the period extension rate after structural failure and the epicenter distance, the main aftershock PGA ratio, site type, duration, elastic fundamental period, ductility coefficient, stiffness degradation rate, cumulative damage and other factors has been emphatically studied, and the final period extension rate estimation formula is given.

The structural failure factor is established according to the structural dynamic equation by Gunawan^12^. The structural failure factor iterative calculation method is constructed using the high-order Runge–Kutta method, and the sensitivity assessment of the structural failure factor was verified using single-degree-of-freedom and double-degree-of-freedom system.

Gunawan et al.^13^ applied Euler–Bernoulli beam theory to construct a structural damage assessment formula that taking structural natural vibration period as the dominant factor.

According to the above research literature, it is known that necessary research has been carried out in the calculation of structural fundamental period and structural damage assessment based on periodic changes. However, most of the existing period calculation formulas are based on empirical formulas, and most of the formulas use the height of the structure or the number of floors to directly estimate the fundamental period of the structure. Although a fewer independent variables can increase the convenience of formula application, it also sacrifices the accuracy of the formula for calculating the fundamental period of complex and diverse structures, which is unfavorable for structural damage assessment based on periodic changes. At the same time, the existing research results mostly focus on the structural period extension ratio after the structure is completely destroyed. While the relative research results on the period change interval corresponding to different damage levels including slight damage, moderate damage, severe damage, and destroy are few. In this paper, aiming at the shortcomings of the existing research work, the mechanical analysis of the generalized single degree of freedom system is carried out by using the structural dynamics theory, and the fundamental period estimation formula of the structure based on displacement is obtained. Using the direct coupling relationship of structural damage, structural displacement response, structural stiffness degradation and structural periodic change, the estimation interval of structural periodic change factor corresponding to different failure levels is established. Finally, the seismic damage example is used to verify the research method.

## Fundamental period calculation formula

As a complex multi-degree-of-freedom structural dynamic system, the dynamic characteristics of the building structure are closely related to the structural stiffness and mass distribution. For the building structure with uniform mass and stiffness distribution, it can be simplified as a generalized single-degree-of-freedom system in the analysis of structural dynamic characteristics. That is, assuming that the lateral displacement of the building structure is in a single deformation form under external loads such as earthquake and wind load, the structure has only one degree of freedom in the sense of structural dynamics. For the generalized single degree of freedom system, the generalized mass *M*_z_ and the generalized stiffness *K*_z_ associated with the single degree of freedom should be determined first in the process of calculating the natural vibration period^14^.

**Figure 1:**
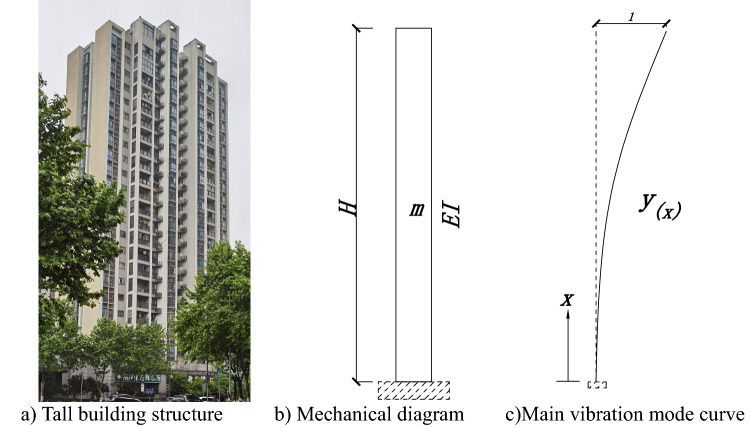
Mechanical diagram and main vibration mode curve diagram of building structure.

For the building structure with bending deformation, the calculation diagram and the main vibration mode curve are shown in Fig. 1.

Figure 1c shows the main vibration mode, assuming its shape function is Eq. (1).

1 $$y_{(x)} = - \frac{1}{3} + \frac{4x}{{3H}} + \frac{1}{3}\left( {1 - \frac{x}{H}} \right)^{4}$$

where, H is structural total height.

Then Eqs. (2) and (3) can be obtained.

2 $$M_{Z} = \int_{0}^{H} {\overline{m} y^{2} \left( x \right) d _{x} } = 0.257mH$$

3 $$K_{Z} = \int_{0}^{H} {qy\left( x \right) d_{x} } = 3.2\frac{EI}{{H^{3} }}$$

So the formula for calculating the fundamental period of structure is Eq. (4).

4 $$T _{1} = 2\pi \sqrt {\frac{{M_{Z} }}{{K_{Z} }}} = 2\pi \sqrt {\frac{{0.257mH^{4} }}{3.2EI}}$$

Let *w* = *mg* and gravity acceleration *g* = 9.8 m/s^2^. And let $\frac{{wH^{4} }}{8EI} = \Delta_{n}$ is the vertex displacement of the structure under uniform load *w*, then Eq. (5) is obtained.

5 $$T _{1} = 2\pi \sqrt {\frac{{0.257mH^{4} }}{3.2EI}} = 2\pi \sqrt {\frac{0.257 \times 8}{{3.2g}}} { \cdot }\sqrt {\frac{{wH^{4} }}{8EI}} = 1.607\sqrt {\Delta_{n} }$$

## Theoretical hypothesis of structural damage assessment

**Figure 2:**
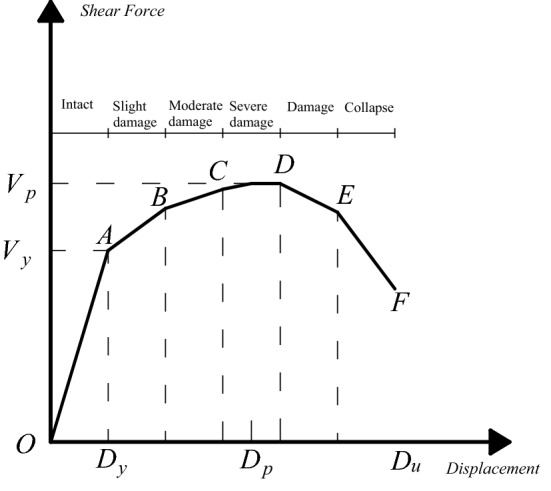
Force–displacement curve of reinforced concrete structure.

For reinforced concrete structures, the lateral load–displacement relationship is shown in Fig. 2.

It can be seen from Fig. 2 that when the deformation curve of the structure under external load is in the O–A stage, it is considered that the structural stiffness is equal to the initial stiffness in the elastic stage, and the structure is basically intact. When the curve is in the A–B stage, the structural stiffness degenerates, the fundamental period becomes longer, and the structural failure state is slight damaged. When the curve is in the B–C stage, the structural stiffness is further degraded, the fundamental period continues to grow, and the structural failure state is moderate damage. When the curve is in the C–D stage, the structural stiffness continues to degenerate, and the structural failure state is serious damage. When the curve is in D–F stage, the structure collapses. In short, in the process of stiffness degradation, the structural displacement response and the structural fundamental period are increasing.

The failure state of the structure under horizontal load is usually measured by the change of inter-story displacement angle. Taking reinforced concrete frame structure as an example, Table 1 lists the corresponding relationship between common displacement angles and different failure states.

**Table 1:** Seismic damage classification standard of reinforced concrete frame structure.

References	Intactness-slight damage	Slight damage-moderate damage	Moderate damage-severe damage	Severe damage-destroy
Chinese seismic design code^15^	1/550	1/250	1/120	1/60
FEMA273^16^	–	1/100	1/50	1/25
Vision 2000 ^17^	1/500	1/200	1/67	1/40
ATC40 ^18^	–	1/100	1/50	1/33
Lu Xilin^19^	1/500	1/300	1/150	1/50

Since FEMA273 and ATC40 code adopt the one-stage design method to check the seismic action, the structural deformation in elastic stage is not directly constrained. Through the comparative analysis of 80 typical RC structures, Han et al.^20^ considered that the limit value of elastic inter-story displacement angle of RC structure should be 1/500. For the convenience of analysis, the elastic inter-story displacement angle corresponding to FEMA273 and ATC40 code is set as 1/500 in this paper.

According to Eq. (1), when the structure is in elastic state, that is, assuming that the structure is in basically intact state, the maximum inter-story displacement angle of the structure corresponding to the fundamental period *T*_0_ is approximately equal to $\delta_{e,n} = \frac{{qH^{3} }}{6EI}$, and the corresponding maximum displacement is $\Delta_{e,n} = \frac{{qH^{4} }}{8EI}$, which can be considered as $\delta_{e,n} = \frac{4}{3H}\Delta_{e,n}$.

Define *λ* as the structural damage factor, namely

6 $$\lambda { = }\frac{{\delta_{x} }}{{\delta_{y} }}{ = }\frac{{\Delta_{x}^{{}} }}{{\Delta_{y} }}$$

where, $\delta_{x}$ is the inter-story displacement angle of after structural damage, $\delta_{y}$ is the inter-story displacement angle of in the yielding state, $\Delta_{x}^{{}}$ refers to the corresponding vertex displacement after structural damage, and $\Delta_{y}^{{}}$ is refers to the corresponding vertex displacement in the yielding state.

The damage state of the structure is determined according to Eq. (6). According to the value of *λ*, the damage state of the structure is determined, as showing in Table 2.

**Table 2:** Structural damage factor interval for different damage states.

References	Elastic threshold	Slight damage	Moderate damage	Severe damage	Destroy
Chinese seismic design code	1/550	1 ≤ λ < 2.2	2.2 ≤ λ < 4.58	4.58 ≤ λ < 9.17	9.17 ≤ λ
FEMA273	1/500	1 ≤ λ < 5	5 ≤ λ < 10	10 ≤ λ < 20	20 ≤ λ
Vision 2000	1/500	1 ≤ λ < 2.5	2.5 ≤ λ < 7.46	7.46 ≤ λ < 12.5	12.5 ≤ λ
ATC40	1/500	1 ≤ λ < 5	5 ≤ λ < 10	10 ≤ λ < 15.15	15.15 ≤ λ
LU Xilin	1/500	1 ≤ λ < 1.667	1.667 ≤ λ < 3.333	3.333 ≤ λ < 10	10 ≤ λ

For the computed results of *λ* to different seismic damage classification standard as showing in Table 2, the interval distribution under different damage states is obviously different. Therefore, the structural damage factor interval of different damage states should be verified according to the earthquake damage examples.

The Eq. (5) shows that the fundamental period of the structure is related to the structure vertex displacement under specific lateral horizontal load, as shown in Eq. (7).

7 $$\Delta_{n} { = }\left( {0.622 \times T} \right)^{2}$$

Structural damage factor λ can be transformed to Eq. (8).

8 $$\lambda { = }\frac{{\Delta_{x}^{{}} }}{{\Delta_{y} }}{ = }\frac{{\left( {T^{\prime}_{1} } \right)^{2} }}{{\left( {T_{1} } \right)^{2} }}$$

where, $T^{\prime}_{1}$ is the fundamental period of the structure suffered by seismic action.

Combined with the relevant limits in Table 2, Eq. (8) can be directly applied to structural damage assessment.

## Verification of the seismic damage examples

**Figure 3:**
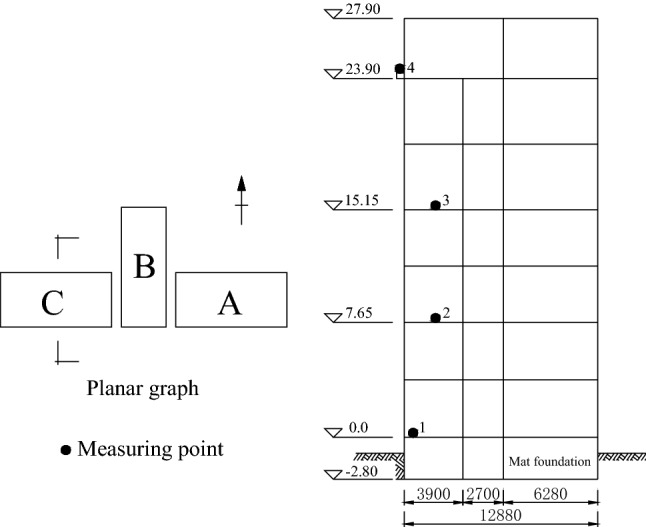
Section and measuring point layout of the building A.

**Figure 4:**
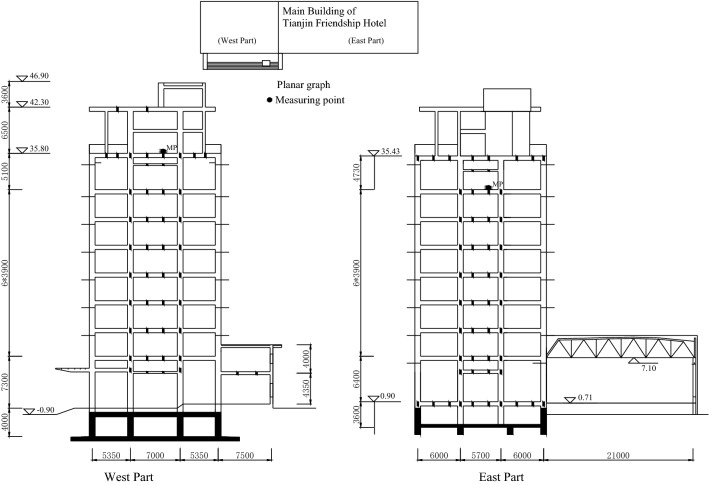
Section and measuring point layout of the building B.

After the Tangshan M7.8 earthquake on July 28, 1976 in China, most areas of Tianjin suffered an intensity of 8 degree. The inpatient department building of Tianjin Hospital (hereinafter building A) and the Tianjin Friendship Hotel building (hereinafter building B) in Tianjin City are affected by strong earthquakes^21^. The fundamental periods of the structure before and after the earthquake were measured by the seismic receiver installed in two buildings. The section and measuring point layout of the two structures are detailed in Figs. 3 and 4. The basic situation, seismic damage characteristics and fundamental period changes of the two structures are shown in Table 3. ‘BE’ in Table 3 represents before the earthquake, and ‘AE’ in Table 3 represents after the earthquake.

**Table 3:** Overview of two buildings in Tianjin, China.

Serial number	Building name	Storey number	Height	Seismic precautionary intensity	Encountered intensity	Damage level	Fundamental period		λ
							BE	AE	
S1	Building A	8	33.4	Non-fortification	8 degree	Slight damage	0.55	0.61	1.23
S2	East Section of building B	8	37.4	7 degree	8 degree	Slight damage	0.5	0.85	2.89
S3	West Section of building B	11	47.3	7 degree	8 degree	Slight damage	0.5	0.67	1.80

The parameter λ in Table 3 is calculated by Eq. (8). According to the calculation results of λ in Table 3 and the actual seismic damage level of the structure, the damage state assessment interval determined by different seismic damage classification standards in Table 2 is compared. It is shown that the damage state assessment interval determined by FEMA273 and ATC40 is reasonable, and the damage state assessment standard in Table 2 is basically in line with the actual seismic damage performance. Therefore, the structural seismic damage assessment method established in this paper based on the fundamental period change of structure has good assessment results and high engineering application value.

Zembaty et al.^22^ carried out shaking table tests on a full-scale reinforced concrete frame structure and monitored the fundamental periods of the two structures before and after failure. The monitoring results and damage factor calculation results are shown in Table 4.

**Table 4:** Calculation results of the frame.

The damage state		Elastic threshold	Slight damage	Slight damage	Slight damage	Moderate damage
Frame	Direction X	1.29	1.59	2.52	2.99	3.71
	Direction Y	1.05	1.33	1.90	2.21	2.76
*λ*			1.51	3.83	5.38	7.24
			1.61	3.29	4.44	6.95

The calculation results in Table 4 show that the seismic damage state of building determined by Formula 8 and Table 2 is basically consistent with the test results. At the same time, the damage factor intervals corresponding to different damage state determined by FEMA273 and ATC40 have better matching results.

## Conclusions

In this paper, the repaid seismic damage assessment of overall building structures based on fundamental period is studied. The results are summarized as follows.

1. The fundamental period calculation formula of generalized single-degree-of-freedom system based on fixed-point displacement calculation is established by using the conversion mass method, selecting the appropriate vibration mode function and the distribution mode of horizontal seismic action.
2. According to the relationship curve between force and displacement of reinforced concrete structure, the mapping relationship between fundamental period of structure and structural damage factor is established with displacement response as intermediate variable.
3. Combined with the seismic damage assessment standard of building structure based on inter-story displacement angle, the seismic damage assessment criterion of building structure with fundamental period change is established.
4. Seismic damage examples are used to verify the established seismic damage assessment method of building structures. The verification results show that the established method in this paper has good assessment results and high engineering application value.

The rapid assessment method for seismic damage of the overall structure established in this paper can provide technical reference for the safety identification and health monitoring of building structures. Considering that the change of the fundamental period of the structure is insensitive to the local damage of the structure, the influence of the high-order period on the seismic damage assessment of the structure should be studied in the future.

## Data Availability

All data generated or analysed during this study are included in this published article.
